# Plant In Vitro Systems as a Sustainable Source of Active Ingredients for Cosmeceutical Application

**DOI:** 10.3390/molecules25092006

**Published:** 2020-04-25

**Authors:** Andrey S. Marchev, Milen I. Georgiev

**Affiliations:** Laboratory of Metabolomics, Department of Biotechnology, The Stephan Angeloff Institute of Microbiology, Bulgarian Academy of Sciences, 139 Ruski Blvd., 4000 Plovdiv, Bulgaria; andrey.marchev@yahoo.com

**Keywords:** plant in vitro systems, cosmeceuticals, bioreactor cultivation, anthocyanins, metabolic engineering, gene expression

## Abstract

Cosmeceuticals are hybrids between cosmetics and pharmaceuticals which are being designed for a dual purpose: (1) To provide desired esthetical effects and (2) simultaneously treat dermatological conditions. The increased demand for natural remedies and the trends to use natural and safe ingredients resulted in intensive cultivation of medicinal plants. However, in many cases the whole process of plant cultivation, complex extraction procedure, and purification of the targeted molecules are not economically feasible. Therefore, the desired production of natural cosmetic products in sustainable and controllable fashion in the last years led to the intensive utilization of plant cell culture technology. The present review aims to highlight examples of biosynthesis of active ingredients derived through plant in vitro systems with potential cosmeceutical application. The exploitation of different type of extracts used in a possible cosmeceutical formulation, as well as, their activity tested in in vitro/in vivo models is thoroughly discussed. Furthermore, opportunities to manipulate the biosynthetic pathway, hence engineering the biosynthesis of some secondary metabolites, such as anthocyanins, have been highlighted.

## 1. Introduction

Plants are distinctive in their broad range of chemical structures synthesized and are the biggest source of primary and secondary/specialized metabolites (SMs) used in food, pharmaceutical, and cosmetic products [1]. During the recent years the cosmetic industry is performing a profound screening for bioactive molecules with indicative health benefits for humans. These ingredients are still preferred to be extracted from natural sources, such as herbal or medicinal plants [2]. This strategy led to the appearance of a new term, “cosmeceutical”, used to describe a hybrid substance designed to serve as a dual purpose: provide desired esthetical effect (as a cosmetic) and to treat dermatological conditions (as a pharmaceutical) [3]. The same compounds, which protect plants from environmental stresses, such as salinity, UV, drought and extreme temperature amplitudes, can have similar protective effects on human skin, via the activation of basic protective signaling mechanisms conserved in the eukaryotic cells [4]. The applied ingredients in cosmeceuticals include phytochemicals, such as phenylpropanoids and their derivatives (flavonoids, phenolic acids, tannins, glycosides, and lignins), terpenes (isoprenoids, terpenoids), nitrogen containing compounds (alkaloids and hetrocyclic aromatics), vitamins, essential oils, amino acids, peptides, and sugars, and activate diverse signaling pathways in human skin cells leading to the up-regulation of specific aging-associated genes, thus protecting the cells from stress factors [5,6]. Therefore, phytochemicals with desired properties, such as antioxidant, anti-aging, antimicrobial, anti-inflammatory, anti-wrinkling, skin whitening, and photoprotective activities are of significant interest for the cosmetic industry [7].

At present the cosmetic industry accounts for 412 billion Euro in annual sales, of which Japan has the biggest market value of about 5.6–7.4 billion Euro, followed by USA of approximately 4.6–5.6 billion Euro and EU with 2.8–4.6 billion Euro, making it highly desirable and competitive field [3,5]. For instance, the global market of anthocyanins is expected to reach 358.7 million Euro in 2021 [8], while that of carotenoids (1.38 billion Euro in 2017) has a forecast peak of 1.85 billion Euro in 2022. The market value of the most consumed carotenoid, β-carotene increased from 241.7 million Euro in 2010 to 309 million Euro in 2018 [9]. According to these observations the global market of phytochemicals will continue to grow exponentially, and might result in discrepancy between their demand and availability [10].

Over the past 10 years an increasing interest has been observed in plant cell culture extracts, which contain a wide range of primary and SMs, possessing multiple beneficial activities for skin- and hair care [11]. Alternatively, plant biotechnology techniques, and in particular plant in vitro systems, offer a reliable and sustainable platform for bioproduction of plant derived SMs. Major advantages of plant cell culture is their totipotency and the possibility to produce the desired SMs in high yield and consistency, independent of the climate conditions with much shorter production cycles. The process is performed under controlled aseptic conditions as defined by the good manufacturing practices, thus assuring safe end products that lack genetic or environmental contaminations [10,12]. Plant in vitro systems propose immense potential to increase the SMs production via genetic manipulation of the biosynthetic pathways or optimization of the cultural conditions, e.g., addition of precursors, elicitors, and nutrient medium optimization. Product extraction and purification is also found to be easier as compared to the whole plants [13]. Plant in vitro systems can be used as a model to unravel biosynthetic pathways and investigate the factors that modulate these pathways, increasing the possibility for establishment of commercial processes for bioproduction of high-value SMs [14]. This biotechnological tool might also be applied in the regeneration of transgenic plants with boosted anthocyanin content, for instance [15].

Because of the more simplified bioreactor design, less cycle of production and uniform growth, plant cell suspension culture are still preferred system for the bioproduction of SMs at lab- or large-scale size. However, due to their heterogeneity, weak growth and inconsistent yield of natural products (NPs) are observed often [1]. Therefore, in some cases hairy roots as differentiated cultures, considered as more genetically stable with higher and consistent NPs accumulation are used [16]. The cambial meristematic cells (CMCs) can further help to avoid the mentioned issues for suspension cultures. The CMCs are undifferentiated cells and function as plant stem cells, because of their procambium meristematic cells origin and therefore do not dedifferentiate. These cells have been used by the “Unhwa Corp.” company (Korea) for the development of CMC-based suspension cultures of *Taxus cuspidata*, *Ginko biloba,* and *Solanum lycopersicum* with cosmetic application [17]. Important aspect in cosmeceuticals is to clarify the term “plant stem cells”. Indeed, in almost all cases the cosmetic products contain stem cell extracts, not live stem cells and this term is sometimes equal to active molecules extracted from plant cell suspensions or hairy roots [18].

The present review attempts to summarize the successful examples of biosynthesis of active ingredients with cosmeceutical application based on plant in vitro systems. The utilization of different type of extracts used in a possible cosmeceutical formulation, as well as, their activity tested in in vitro and in vivo models is discussed. Moreover, a detailed presentation of the anthocyanins biosynthetic pathway, including the possibilities to enhance their biosynthesis in plant in vitro systems is given.

## 2. Plant In Vitro Systems Derived Cosmetic Ingredients

Since plant in vitro systems are supreme and continuous source of active ingredients, they are point of intense interest by the cosmetic industry. The metabolites produced differ in their chemical nature, stability, and solubility. Due to this reason, it is of critical importance to use appropriate extraction procedure and solvents in order to extract the compounds of interest. The most frequently used extracts in cosmetics are plant cell wall extracts, hydrosoluble (extracted with glycerol), liposoluble (extracted with oils), extracts rich of desired metabolites and extracts with reduced content of potentially toxic compounds [19]. In addition to that, powdered extracts (with maltodextrin), liposomes, nanoemulsions, and suspensions are also available [20].

Whole plant cell wall extracts are usually rich in amino acids, peptides, proteins, and sugars. Small peptides, especially those rich in the amino acids like glycine, proline, and hydroxyproline induce diverse defense response mechanisms in mammalian skin. Plant cell wall proteins are similar to collagen in animals and are preferred ingredients for cosmetic products, since they are much safer for the humans. Moreover, amino acids are directly responsible for skin moisture effect as they are the main component of the natural moisturizing factor (NMF) [4]. Whole plant cell wall extracts contains sugars (hexoses, pentoses, and oligosaccharides), which can also promote the anti-aging effect of the skin, enhance its hydration and suppress inflammatory processes [4,6]. A novel plant “biostimulator” extracted from the whole cell walls of *Nicotiana* spp. represents a mixture of peptides and sugars where protein sugar fraction was found 12.88%. Peptide analysis indicated the presence of internal sequences of proline or isoleucine/leucine/hydroxyproline, among which the most abundant were glycine (16%) and proline (8.5%). The sugar fraction was composed of mostly hexoses (galactose, glucose, and mannose) and pentoses, such as, arabinose. This peptide/sugar mixture was able to increase collagen synthesis and stability, enhance DNA protective and repair functions and attenuate oxidative changes in cultured human cells [4].

Hydro-soluble extracts contain amino acids, peptides and different classes of polyphenols, such as phenolic acids, flavonoids, and anthocyanins. The hydro-soluble extract of *Lycopersicon esculentum* was found rich in antioxidant compounds and metal-binding peptides, such as phytochelatins and metallothioneins. Phytochelatins have a structure characterized by the repetition of glutamate/cysteine, which is further bound to glycine, while metallothioneins are composed of 60–80 amino acids and contain 9–16 cysteine residues. Among these phenolic compounds, the most abundant were chlorogenic, coumaric acids and rutin, which are known for their anti-aging effects. This water-soluble extract exhibited skin protection activity towards heavy metal toxicity by preventing DNA integrity and collagen degradation [21].

An example for a liposoluble extract is the one obtained from *Rubus ideaus* cell suspension that contains phenolic acids and wide range of fatty acids. The oil-soluble extract contained several types of fatty acids, such as palmitic, stearic, oleic, linoleic, and α-linolenic acid. These fatty acids are also the primary components of the epidermal protective hyrdrolipic film and have key role in the hydration and reparative responses in the skin. Several long chain fatty acids with anti-inflammatory activity, such as arachidic and arachidonic acids, were also identified. The major phenolic compounds were coumaric, ferulic acids, and kaempferol. The extract revealed good potential to be used as cosmetic active ingredient, as it improves the skin hydration and moisturization capacity, skin lipid production and collagen synthesis [22]. However, the disadvantage of water- and oil-soluble extracts is that they are based on the targeted extraction of closely-related bioactive compounds, thus cannot show the complete health benefits of whole cell extracts. That is why the Switzerland company Mibelle AG Biochemistry developed a liposomal extract from *Malus domestica* cell suspension, which contained the water- and fat-soluble components of the cell extract simultaneously [23].

Plant in vitro systems are frequently used to obtain extracts enriched in specific targeted molecules obtained during their cultivation in submerged conditions. For example phytoene (with UVB protection properties) [24], resveratrol, oxyresveratrol, and milberoside A (possessing tyrosinase inhibitory and whitening effect) [10,25,26], verbascoside (with anti-inflammatory and wound healing properties) [27] and arbutin (with tyrosinase inhibition and whitening activity) were produced using plant in vitro systems [28].

The extracts obtained from plant in vitro systems used in cosmetics are considered as safe, and so it is important that they do not contain substances with potential toxic or allergic effect. Such a case is the content of pyrrolizidine alkaloids identified in the plant *Lithospermum officinale*. However, the suspension culture from this plant species did not contain these toxic alkaloids [29]. Similarly, an advantage of the extract by *L. esculentum* is the absence of the alkaloids α-tomatine and dehydrotomatine, which might cause allergic reactions [21].

## 3. Small- and Large-Scale Production of Cosmetic Ingredients through Plant In Vitro Systems

Bioreactor-based cultivation and scaling-up into large-scale production is the final stage for the biosynthesis of the desired SMs. Plant in vitro systems have the potential to provide a low-cost production of diverse plant-derived products. However, their large-scale production is challenged by several biological barriers, including cell size, low metabolite yield, cell heterogeneity and genetic instability, and technological barriers, such as shear stress sensitivity, intensive mixing and aeration, cell aggregation, foaming and adhesion of the cells [30,31]. For preventing the slow growth, which results in low and unstable productivity, bioreactor design and cultivation parameters must be optimized and ensure adequate mixing, gas exchange and homogeneity [32]. The development of a biotechnological process depends also on the biological significance of the secondary metabolites, their application and market value, e.g., the global market value of anthocyanins was 270 million Euro in 2004 and is expected to reach 358.7 million Euro in 2021. The price of the purple anthocyanins, which have higher health beneficial value, is at least 110 Euro/mg [8].

In this section, we have selected examples of bioreactor-based production of SMs intensively used as cosmetic ingredients, such as anthocyanins, phenolic acids, flavonoids, tocopherol, and verbascoside, including the optimization procedures to enhance their biosynthetic capacity, such as optimization of inoculum concentration, agitation, aeration, bioreactor design, temperature, light irradiation and others. The optimum inoculum concentration for the cultivation of the transgenic *Nicotiana tabacum* cv. *Samsun* in a 2-L stirred tank bioreactor was established to be 3%, while the optimum temperature was 23 °C. The used suspension was a mutant version in bHLH Delila protein (*Am*Del*) in combination with MYB Rosea1 (*Am*Ros1). In this combination the cell suspension produces trace amounts of pelargonidin 3-*O*-rutinoside and dominantly cyanidin 3-*O*-rutinoside with red color, much as 90 mg/L under the optimized conditions [8]. Examples of biomolecules generated via bioreactor-based cultivation are presented in Table 1.

An important parameter used to characterize bioreactor cultivation is the oxygen mass transfer coefficient k_L_a (1/h), which depends on the shear stress rate, aeration rate, agitation speed, nutrient medium composition, surface active agents, cell concentration and sparger type [32]. In most of the cases, the aeration rate lies between 0.033 and 0.1–0.2 vvm [33], while the agitation speed usually varies between 50 to 100 rpm [32,37]. These parameters normally ensure the dissolved oxygen to be between 10% to 30%, which is enough air saturation [32]. The cultivation of *Vitis vinifera* (L) cv Gamay Fréaux var. Teinturier was performed in a 2-L Rushton turbine stirred bioreactor at 75 rpm. To ensure sufficient oxygen supply during the whole process the initial aeration rate (0.075 vvm) was increased to 0.15 vvm at day 5 of the cultivation due to the increased cell density. Optimization of media composition, such as increases of sucrose and phosphate (2.2 mM) concentration and half reduction of ammonium and magnesium concentrations, resulted in shorter cell cycles and increased production of anthocyanins (387 mg/L) for 12 days, while the cultivation process in the flasks continued for 14 days [33]. The optimized nutrient medium (5% sucrose and 1.5 mg/L 2.4-dichlorophenoxy acetic acid) doubled the production of squalene to 5.5 mg/g DW during the cultivation of *Santalum album* in 7-L stirred tank bioreactor compared to the flask cultivation [44]. Optimization of inoculum density (20 g/L), sucrose (30 g/L), and casein (500 mg/L) concentrations permitted the accumulation of 53.80 mg/L resveratrol from *V. amurensis* cell suspension culture in a 3-L balloon type bioreactor [45]. The ratio between NH_4_^+^ and NO_3_^-^ of 5.25 mM was the optimum to produce 12.70 μg/g DW chlorogenic acid when *Eleutherococcus koreanum* Nakai adventitious roots were cultivated in a 3-L bulb type balloon bioreactor [41]. The reduction of the Murashige and Skoog medium to 1/4 resulted in an increased production of chlorogenic acid (24.68 mg/L) in comparison to full strength medium (11.47 mg/L) when the adventitious roots of *El. koreanum* Nakai were cultivated in the same bioreactor system [46]. An air flow rate of 80 mL/min during the cultivation of *Vitis vinifera* cv. Bailey alicant A in an airlift bioreactor was the optimum for production of 33 mg/L anthocyanins after 13 days. Flow rates above this value (160 mL/min) resulted in significant shear stress, while lower values (40 mL/min) resulted in cell sedimentation and in both cases leading to lower anthocyanin content [35]. Bioreactor modifications can be used to improve the k_L_a coefficient and product biosynthesis rates. During the cultivation of *P. frutescens* in a 2.6-L stirred tank bioreactor the original ring sparger (with pore size 500 μm) was replaced with a sintered glass one (pore size between 30 and 80 μm). The smaller bubbles increased not only the k_L_a value from 9.9 to 23 L/h, but also the anthocyanin content from 480 mg/L to 1650 mg/L [36]. Another option to increase the mass transfer is to control the bubble size in the culture medium through the addition of 0.8% carboxymethyl cellulose. The increased viscosity produced smaller bubbles uniformly distributed in the vessel volume, resulting in almost double anthocyanin production in comparison with the medium without carboxymethyl cellulose [35]. Light is also an important parameter that influences biomolecule production, particularly anthocyanins. The continuous exposure to fluorescent light (5000 lux) resulted in the production of 1200 mg/L anthocyanins (12% cyanidin-3-glucoside, 68% peonidin-3-glucoside and 8% malvidin-3-glucoside) during the cultivation of *V. vinifera* (L) cv Gamay Fréaux var. Teinturier in 20-L stired tank bioreactor [34]. The increase in light intensity up to 27 W/m^2^ doubled the production of anthocyanins in an airlift (3.0 g/L) and stirred tank bioreactor (2.9 g/L) during the cultivation of *P. frutescens* cell suspension [34].

Due to the variable cell suspension growth pattern and inconsistent yield of NPs, shoot or hairy root cultures have been also used for the production of SMs. They frequently require bioreactor design, which creates low hydrodynamic stress environment, such as the nutrient sprinkle. It has some benefits, such as better control of growth conditions, optimal supply of nutrients and optimal gas environment [16]. The biosynthesis of rosmarinic acid, a molecule with high antioxidant capacity [47], was more favorable in the hairy roots of *S. officinalis* (477.13 mg/L), than in the shoot cultures (59.04 mg/L) cultivated in nutrient sprinkle bioreactor [38]. Accumulation of rosmarinic acid of 17.90 mg/g DW was obtained from *Dracocephalum forrestii* shoots when cultivated in a nutrient sprinkle bioreactor with pump operation for 25 s and at 2.5 min breaks [16]. The accumulation of rosmarinic and chlorogenic acids was 448 mg/L and 302 mg/L, respectively during the cultivation of *Leunorus sibiricus* transgenic hairy roots in a 5-L nutrient sprinkle bioreactor. The transgenic roots contained the AtPAP1 transcriptional factor (*Arabidopsis thaliana* transcriptional factor), which regulates genes from the phenylpropanoid pathway [39]. Adventitious roots are also used for the biosynthesis of plant-derived secondary metabolites. They are stable production platforms, showing fast growth rates in the presence of plant growth regulators without the insertion of foreign genes [48]. Approximately 461 mg/L caffeic acid derivatives were accumulated during the co-cultivation of *Echinacea pallida* and *E. purpurea* adventitious roots in 5-L balloon type bioreactor [49]. Column bioreactors offer lower shear stress conditions than the stirred tank bioreactors and this was clearly demonstrated by the cultivation of *Harpagophytum procumbens* cell suspension in a stirred tank and glass-column bioreactor with pulsed aeration. The content of verbascoside was 445.44 mg/L and 496.3 mg/L in the stirred tank and the column bioreactor, respectively [42]. In the case of verbascoside, cell suspensions are superior source of this molecule compared to differentiated cultures. The cultivation of *Scutellaria alpina* shoots in a nutrient sprinkle bioreactor resulted in 11.4 mg/L production of verbascoside [43].

One of the effective approaches to increase the SMs biosynthesis is the use of elicitors. The addition of 0.2 mM methyljasmonate (MeJa) during the exponential phase of the bioreactor cultivation of *V. vinifera* cv. *Chasselas×Vitis berlandieri* resulted in 209 mg/L resveratrol production, while this molecule was not produced without the elicitor [30]. Resveratrol, ε-viniferin, pallidol, and labruscol biosynthesis in *V. labrusca* cell suspension was stimulated by the addition of cyclodextrines (13 mM) [37]. Fed-batch processes along with elicitation enhanced the production of resveratrol when compared to the batch cultivation of *V. labrusca*. The content of resveratrol reached maximum of 66 mg/L on after 12 days of cultivation [32]. Elicitation with 50 μM MeJa resulted in 78.22 mg/L production of chlorogenic acid in a 3-L air lift bioreactor [40]. The same amount of MeJa resulted in the production of 54.17 mg/g DW total phenolics during the cultivation of *Polygonum multiflorum* in 5-L balloon type bioreactor after 21 days of cultivation, which seems to have a potential and for large-scale cultivation. In comparison, the mother plant had 100.86 mg/g DW but after 5 years of cultivation [50].

Despite the given examples of bioreactor-based production of plant-derived SMs there is still a limited number of large-scale cultivation processes. Some examples include the cultivation of *Hypericum perforatum* adventitious roots in 500-L balloon type airlift and horizontal drum type bioreactor. The airlift bioreactor was more suitable for biomass and SMs accumulation in comparison with the drum type. The accumulated biomass was 6.3 kg, while the chlorogenic acid content reached up to 1.3 mg/g DW [51]. The *Echinacea angustifolia* adventitious roots produced 22.06 mg/g DW total phenolics, 5.77 mg/g DW total flavonoids and 10.63 mg/g DW caffeic acid derivatives when cultivated in a 500-L balloon type bioreactor the [48]. Large-scale cultivation process in 500-L balloon type and 1 000-L drum type bioreactor was developed for the biosynthesis of chlorogenic acid from adventitious roots of *E. purpurea*. After 50 days of cultivation the content of chlorogenic acid was 4.9 mg/g DW and 4.4 mg/g DW in the drum type and balloon type bioreactor, respectively [52]. An example for large-scale anthocyanin production was demonstrated by the cultivation of *Aralia cordata* cell suspension in 500-L stirred tank bioreactor operating at 25 °C, agitation at 30 rpm and 0.2 vvm aeration rate [53]. Several cosmetic companies use large-scale cultivation of plant in vitro systems for the production of anthocyanins from *Euphorbia milli* and *Aralia cordata* in rotary culture system (Nippon Paint Company, Tokyo, Japan), arbutin from *C. roseus* (Mitsui Petrochemical Industries, Tokyo, Japan), rosmarinic acid from *Coleus blumei* (Nattermann, Vettelschoss, Germany), taxol from *Taxus brevifolia* and *T. cuspidata* in 75 000-L impeller driven and air lift reactor systems (ESCAgenetics Phyton Inc., Wisconsin, WI, USA; Nippon Oil Company, Tokyo, Japan and Samyang, Genex Co. Ltd., Seoul, Korea) and some others reviewed in [53,54].

Whole cell extracts are often preferred for cosmetic applications, since they contain both hydro- and oil-soluble cell components and might have complex beneficial effects on human skin. Examples of large-scale production is the cultivation of *Rubus chamaemorus* cell suspension in a 300-L stirred tank bioreactor operating at 25 °C, 50–100 rpm agitation, 15 L/min aeration level and 20% dissolved oxygen. A 14-fold increase in the biomass was observed and the obtained extract consisted of 37% proteins, 195 mg/g sugars (mainly sucrose, glucose and fructose), free (12.7 mg/g) and esterified (16.6 mg/g) fatty acids, respectively. The major esterified fatty acids were α-linolenic, palmitic and linoleic acids and free fatty acids were mainly α-linolenic and palmitic acids. In addition, the extract contained β-sitosterol and α-tocopherol, flavanols, hydroxycinnamic and hydroxybenzoic acids [55]. A pioneer in the development of such complex extracts is the Mibelle AG Biochemistry from Switzerland. Their first product (PhytoCellTec^TM^ Malus Domestica) developed from stem cell extract from the Uttwiler Spätlauber apple, incorporated in lecithin liposomes contained simultaneously, both hydro- and oil-soluble molecules. The cell suspension was cultured in 50 to 100-L wave mixed disposable bioreactors with aeration of 0.1 vvm. [23]. After 20 days of cultivation the biomass production was 25 g/L/day, which is sufficient for development of industrial process [56]. Further, the company developed products, based on extracts from plant stem cells of *V. vinifera* (PhytoCellTec^TM^ Solar Vitis), *Saponaria pumila* (PhytoCellTec^TM^ nunatak^®^) and *Argania spinosa* (PhytoCellTec^TM^ Argan) with anti-wrinkle and skin regeneration activity. Other stem cell extracts that can be mentioned are those from *Zingiber officinalei, Iris pallida*, *Olea europea*, *Hibiscus rosa*, *Camellia sinensis* (Naylos company, France) with anti-age effects on skin [20].

## 4. Metabolic Engineering of Anthocyanins Pathway in Plant In Vitro Systems

Anthocyanins are natural, water-soluble glycosylated subclass of flavonoids, providing a diverse color range from orange, red, purple to blue in fruit plants and crops [57,58]. They fulfill a range of specific and vital roles in plants, such as protection (from ultraviolet irradiation, pathogen attack, photo-oxidative injury, and temperature) and are used to attract pollinators and facilitate seeds dispersal [59,60]. Moreover, anthocyanins possess numerous beneficial health properties for humans, including anticancer, anti-inflammatory, anti-obesity, neuro-, cardio-, and hepato-protective and free radical scavenging activities [61,62].

**Figure 1:**
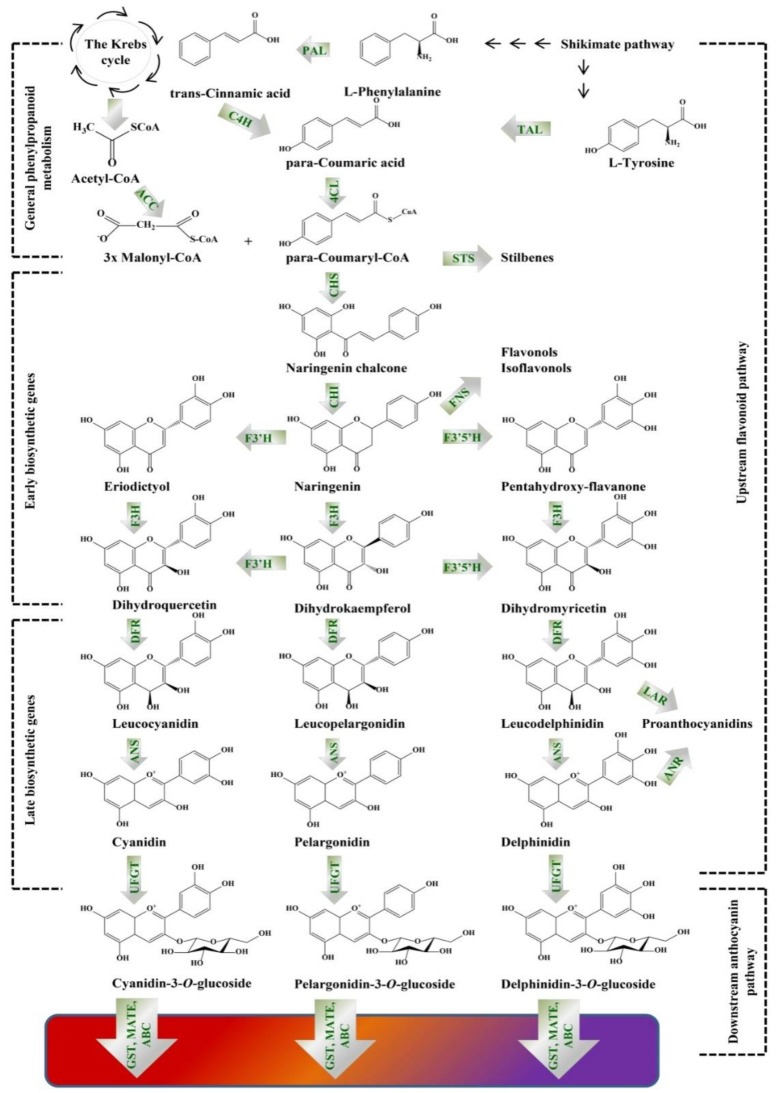
Illustration, presenting the biosynthetic pathway of colored anthocyanins. PAL, phenylalanine ammonia lyase (EC 4.3.1.24); TAL, tyrosine ammonia lyase (EC 4.3.1.23); C4H, cinnamate 4-hydroxylase (EC 1.14.14.91); ACC, acetyl-CoA carboxylase (EC 6.4.1.2); 4CL, 4-coumarate-CoA ligase (EC 6.2.1.12); CHS, chalcone synthase (EC 2.3.1.74); STS, stilbene synthase (EC 2.3.1.95); CHI, chalcone isomerase (EC 5.5.1.6); F3′H, flavonoid 3’-hydroxylase (EC 1.14.13.21); F3′5′H, flavonoid 3´5´-hydroxylase (EC 1.14.13.88); FNS, flavones synthase (EC 1.14.11.22); F3H, flavanone 3-hydroxylase (EC 1.14.11.9); DFR, dihydroflavonol 4-reductase (EC 1.1.1.219); ANS, anthocyanidin synthase (EC 1.14.20.4); LAR, leucoanthocyanidin reductase (EC 1.17.1.3); ANR, anthocyanidin reductase (EC 1.3.1.112); UFGT, UDP-glucose:flavonoid-3-*O*-glycosyltransferase (EC 2.4.1.115); GST, glutathione *S*-transferase (EC EC 2.5.1.18); MATE, multidrug and toxic compound extrusion transporter; ABC, ATP-binding cassette transporter. Phenylalanine is deaminated by PAL to form trans-cinnamic acid that is further converted into para-coumaric acid by C4H. Para-coumaric acid is conjugated with coenzyme A by the enzyme 4CL to obtain para-coumaroyl-CoA. The latter is condensed with three molecules of malonyl-CoA by CHS to generated naringenin chalcone. Subsequently, CHI stereospecifically converts the chalcone to its isomer naringenin. The B ring of naringenin is hydroxylated by F3´H or F3´5´H to produce eriodyctiol or penthahydroxy-flavanone. The (2*S*)-flavanones are next subjected to modification by the conversion of F3H into dihydroflavonols (dihydroquercetin, dihydrokaempferol and dihydromyricetin). Dihydrokaempferol could be directly oxidized by F3´H and F3´5´H to dihydroquercetin and dihydromyricetin. The obtained dihydroflavonols are reduced to colorless leucoanthocyanidins by DFR, which are then oxidized to colored anthocyanidins by the activity of ANS (synonyms leucocyanidin oxygenase: LDOX). Further, glycosylation is performed by UFGT, which occurs in the cytoplasm and produces chemically stable water-soluble pigments.

Anthocyanins are glycosides and acylglycosides of anthocyanidins and the aglycones, flavylium cation (2-phenylbenzopyrilium or 2-phenylchromenylium), that differ in the number of their hydroxyl or methoxyl groups [61,63]. The core structure (flavylium) has a C6-C3-C6 flavonoid skeleton comprising of one heterocyclic benzopyran ring (C ring), one fused aromatic ring (A ring) and one phenyl constituent (B ring). Under the form of cation, anthocyanidins have a positive charge and two double bonds in ring C [59,64]. The hue and color stability of anthocyanidins is directly affected by modifications (hydroxylation and methylation) of their B ring. Thus, cyanidins and pelargonidins are the main precursors of bright red pigments, while delphinidin and its methylated derivatives, as well as, petunidins and malvidins, are sources of dark bluish and purple colors [63]. Anthocyanins biosynthesis undergoes two distinct pathways: the general phenylpropanoid pathway, starting from the amino acid phenylalanine and the flavonoid pathway [15]. This biosynthetic pathway is influenced by its structural and regulatory genes. The biosynthetic pathway has two branches: the upstream basic flavonoid pathway comprised of the early biosynthetic genes (EBGs), and the specific anthocyanin downstream branch, including the late biosynthetic genes (LBGs), as shown on Figure 1. The anthocyanin biosynthesis occurs in the cytoplasm, since the EBGs (chalcone synthase, chalcone isomerase, flavanone 3-hydroxylase, flavonoid 3′-hydroxylase and flavonol synthase) and LBGs (dihydroflavonol 4-reductase, anthocyanidin synthase/leucocyanidin oxygenase and UDP-glucose:flavonoid-3-*O*-glycosyltransferase) are located on the endoplasmic reticulum membranes or in the cytoplasm and further accumulated in the vacuoles [59].

Although anthocyanin biosynthesis is well-known in many plant species, there is still no comprehensive summary about the same in plant in vitro systems. The overall understanding of this pathway might solve one of the biggest challenges in the commercialization of a biotechnological process based on plant in vitro cultivation, such as slow growth, low or no productivity [13]. The anthocyanin biosynthesis pathway has been studied in several plant in vitro model systems, in order to understand and fine-tune its molecular regulation, e.g., *V. vinifera* [65], *A. thaliana* [61], *Daucus carota* [66], *Hypericum perforatum* [67], *Ocimum basilicum* [68], and *Panax sikkimensis* [69] investigating the effect of elicitors [14], precursors [13], plant growth regulators [61], nutrient supply [70,71], and light [72]. In synchronized grape cell cultures, the CHS enzyme [73], as well as, DFR has been recognized as essential contributors for the anthocyanins biosynthesis [57]. The significance of *F3H* gene was proven in purple-colored carrot callus. Knocking out this gene by clustered regularly interspaced short palindromic repeats (CRISPR) together with CRISPR-associated protein 9 (Cas9) resulted in the discoloration of the calli [66]. The red/black color change in muscardine grapes (*V. rotundifolia*) callus was due to induction of the enzymes UFGT and *O*-methyltransferase (OMT; EC 2.1.1.38, synonym anthocyanin *O*-methyltransferase, AOMT), which catalyze the last steps of glycosylation and methylation in the anthocyanins biosynthesis [74]. Elicitors (abiotic or biotic) have been recognized to stimulate defense or stress-induced responses in plants. Some abiotic elicitors such as jasmonic acid (JA), MeJa or salicylic acid (SA) are frequently used to enhance the SMs production in plant in vitro systems, since they act as regulators of defense responses by initiating signals that activate multiple secondary biosynthetic pathways and their relevant genes [75]. For example, MeJa increased the production of total anthocyanins in *V. vinifera L.* cv. *Gamay Fréaux* cell suspension with 1.7-fold, the precursor phenylalanine by 0.7-folds, while the combined application of MeJa (50 mg/L) and phenylalanine (5 mg/L) had a significant synergetic effect increasing the anthocyanin content up-to 5.6-fold [13]. Similarly, the combined treatment of *V. vinivera* cv. Barbera cell suspension with 10 μM MeJa and red light increased the anthocyanin production with 52% [72]. Red light stimulated mostly the production of cyanidin and peonidin in callus cultures of *O. basilicum*, while during dark cultivation the anthocyanin biosynthesis was inhibited [68]. The time-dependent biosynthesis of anthocyanins under 100 μM SA treatment of *V. vinifera* “Cabernet Sauvignon” cell suspension revealed that increased transcription of the EBGs, *VvCHS* and *VvCHI* and *VvDFR* began at 0.5 day after treatment and reached a maximum at day 1. At day 2 the expression of these genes declined, while some of the LBGs, such as *ANS* revealed maximum expression, leading to 73% increase of anthocyanins accumulation [75]. Some inorganic ions, such as Ca^2+^ have also important contribution as secondary signaling messengers in elicitor-induced SMs biosynthesis. During treatment of *V. vinifera* L. cv. Gamay *Fréaux* cell suspension with abscisic acid (ABA) or MeJa, the presence of 10 mM CaCl_2_ resulted in up-regulation of *VvCHS*, *VvDFR,* and *VvUFGT* and down-regulation of *VvFLS* genes, the latest of which catalyzes the flavonols production [76]. The addition of 20 mM MgCl_2_ was beneficial for the accumulation of anthocyanins in *V. vinifera* cv. Gamay Red cell suspension, since Mg^2+^ ions prevented the degradation of anthocyanins through the inhibition of several enzymes, e.g., peroxidases and β-glucosidases [77]. The nitrogen (N), sulfate (S), and phosphate (P) deficiency increased the biosynthesis of anthocyanins in grape cell suspension [65,70,71]. Under S-depletion conditions *CHS*, *DFR, ANS,* and *GST* were up-regulated, while *F3´5´H* and *UFGT* showed no response [70]. During the *p*-deficiency, the grape cell suspension produced dominantly cyanidin, peonidin 3-*O*-glucoside, and coumaroyl derivatives [71]. Auxins might also alter gene expression, which directly or indirectly impacts anthocyanin biosynthesis. Concentrations in the ranges from 2.2 to 27 μM 2.4-dichlorophenoxyacetic acid (2.4-D) and indole 3-acetic acid (IAA) inhibited anthocyanin biosynthesis. During NAA treatment anthocyanin biosynthesis reached a maximum only at 9 μM and decreased afterwards. The same trend of expression was valid for the biosynthetic genes, among which *PAL*, *CHS*, *DFR,* and *ANS* were the most highly expressed [61].

## 5. Extracts (Secondary Metabolites) Activities-Gene Expression and Main Activities

The skin is an organ that has protective functions towards the external environment and its physical integrity is essential to accomplish its functions [11]. Skin aging might happen via two mechanisms: The first termed intrinsic or age dependent, is initiated by several factors, such as telomere shortening, free radicals and antioxidants imbalance and hormonal changes, while the second mechanism—photoaging is due to exposure of the skin to UV solar radiation [20]. Both mechanisms of aging cause inhibition of tissue regeneration, growth and differentiation and mainly occur at the level of dermal connective tissue, resulting in loss of mature collagen, elastin fibers, and hyaluronic acid [78]. Reactive oxygen species (ROS) and heavy metals trigger skin aging via destruction of the antioxidant system, leading to wrinkle formation, melanogenesis and severe damage of biomolecules, such as DNA. [78,79]. Therefore, active phytochemicals that possess the ability to reduce the free radical damage to the skin, and inhibit the activity of elastase, collagenase and hyaluronidase might have beneficial effects in the prevention of skin aging. Several marker activities have been selected to determine the cosmeceutical potential of the plant-derived extracts or pure molecules. For instance, the inhibition of metalloproteinases (MMP), such as MMP1, MMP3, and MMP9 is indicative for prevention of collagen degradation [21]. The whitening activity of extracts is determined by their ability to inhibit the tyrosinase activity, an enzyme responsible for the synthesis of melanin, dopachrome formation and melanogenesis, while the DNA repair mechanisms are indicated by the induction of genes, such as *gadd45α* and *sirtuin-1* (*sirt-1*) [21]. An important aspect for the wound healing process is the skin hydration, determined by the NMF, which comprises a number of substances including ions, small solutes and free amino acids, formed by the breakdown products of filaggrin protein. Indicators for skin hydration are the expression of *aquaporin 3* (*AQP3*) and *filaggrin* (*FLG*) genes. The investigations of these diverse mechanisms require the development of different in vitro and in vivo models. The most frequently used in vitro models comprise cell cultures of keratinocytes (preferably the immortalized human skin cell line HaCaT), primary keratinocytes, epidermal stem cells, and fibroblasts. The in vivo models include animal models of mice, rodents and rabbits and finally clinical trials with humans [80].

The aim of this section is to present the anti-aging, antioxidant, hydration, anti-wrinkling, anti-inflammatory, and wound healing properties of extracts or pure compounds derived from plant in vitro systems. A pronounced activity, indicative for the above-mentioned properties is the antioxidant activity of the plant-derived extracts or pure molecules. The chemical antioxidant assays, such as oxygen radical absorbance capacity (ORAC), hydroxyl radical scavenging capacity (HOSC), and hydroxyl radical averting capacity (HORAC) might be useful but have several limitations concerning the prediction of the antioxidant activity in a biological environment. Therefore, this activity is preferred to be analyzed in cellular assays based on keratinocytes (HaCaT) and fibroblast (HFF and CCD-1112Sk) cell lines. These approaches have the advances compared to the chemical assays, since the influence of several parameters, such as bioavailability, cellular uptake and metabolism is taken into consideration. The ROS are induced by treatment with tert-butyl hydroperoxide (TBHP) or H_2_O_2_ and there are two ways of treatment with the extracts: Pre-incubation of the cells with the extract before the stressor addition and co-incubation of the extracts with the stressor. The pre-incubation treatment gives information about the preventive activity of the extracts, while the co-incubation is closer of a possible therapeutic approach and often seems to be the more effective one [78]. The aqueous cell wall preparation from *Nicotiana* spp. suspension was investigated for its antioxidant capacity using “in vitro” assays, such as total antioxidant capacity (TAC) and ORAC in cultured skin-derived NIH-3T3 fibroblasts. Approximately 85% reduction of ROS was observed when the cells were treated with 3.6 μg/mL extract after their induction with H_2_O_2_. The activity of the extract was higher than that of ascorbate (44 μg/mL) used as a positive control. The observed activity was explained by the direct interaction of the peptides and sugars with H_2_O_2_ or through the activation of the defense response signaling pathways [4].

The 80% aqueous methanolic extract of different *Isodon rugosus* (Wall. ex Benth.) callus cells showed anti-aging and DNA protective activities. Two calli lines distinguished among others, the first one was rich of caffeic and rosmarinic acids, while the second line appeared to produce triterpenoids oleanolic, betulinic, and plectranthoic acids mostly. The pentacyclic triterpenoids were correlated with the elastase, collagenase and tyrosinase inhibitory activity, while the phenolic acids, which were the main contributors to the antioxidant activity correlated with the SIRT-1 (class III deacetylase) activation, hyaluronidase and advanced glycation end-products (AGEs) inhibition [81]. Some of the main activities and gene modulation properties of the analyzed extracts are presented in Table 2.

The effect on melanogenesis of aqueous *C. junos* extract was evaluated on B16F10 melanoma cell line. The tyrosinase activity was inhibited by 25.2% (at 500 μg/mL extract), while the melanin synthesis decreased with 208% (at 50 μg/mL extract) compared to the inhibition caused by arbutin (16.3%), at the same concentration. The skin regeneration activity was tested on fibroblasts cell model. At 500 μg/mL extract concentration 154% fibroblast proliferation was achieved, which was higher than the effect of the recombinant human transforming growth factor-β (TGF-β) at 500 ng/mL, utilized as a positive control. This extract revealed also anti-wrinkle activities evaluated via the increased pro-collagen type I C-peptide in a dose dependent manner. The highest pro-collagen synthesis (1.76-fold higher) was achieved at 500 μg/mL extract concentration. The wound healing effect of the extract revealed 161% increase in the recovery of the wound closure compared to the control and increased the proliferation and migration of the fibroblasts with 15%. The main compound identified and eventually responsible for the observed activities was *p*-hydroxycinnamoylmalic acid [79]. The aqueous cell walls extract (mainly peptides and sugars) of *Nicotiana tabacum* BY2 suspension exhibited DNA protection and repair functions, as well as, increased collagen synthesis and stability in human keratinocytes (HaCaT cell line) and murine fibroblasts (NIH-3T3 cells). Indication for the DNA protective activity was the elevated expression of GADD45α protein and SIRT-1 and SIRT-6 in the fibroblasts after treatment with 150 μM H_2_O_2_. Further, this effect was confirmed by the “single cell electrophoresis assay” (comet assay). The length of the nucleus comet, index of DNA fragmentation was significantly reduced (with 50%), clearly indicating the genomic DNA protection. The peptide/sugar mixture elevated the expression of collagen type I and III genes with 200% and also increased the levels of pro-collagen I and III synthesis 2–3-folds. Beside the effect to induce new collagen production, the same cell wall preparation inhibited the expression of MMP1, MMP3, and MMP9, responsible for the ECM degradation [4]. The hydrosoluble extract of *L. esculentum* cultured stem cells protected the NIH-3T3 fibroblasts and HaCaT keratinocytes collagen degradation and DNA integrity from heavy metal damages [21]. The aqueous extract from *H. syriacus* cell culture stimulated the production of pro-collagen type I and fibronectin with 60% and 16%, respectively and improved the wound healing properties in HaCaT and human derma fibroblasts (HDF), isolated from 48-year-old woman with 50% and 30% when treated with extract concentration of 20 μg/mL. The observed beneficial effects were explained by the hydration properties of the extract, which was able to increase the expression of AQP3 and FLG expression with 20% and 60% respectively, thus contributing to the NMF production and maintenance of skin water balance [80]. An oil-soluble extract of *R. ideaus* suspension (containing phenolic acids and wide range of fatty acids) revealed significant skin hydration and collagen protection activities in in vitro studies, performed on HaCaT cells. The extract was found to improve the skin lipid production through the activation of two enzymes catalyzing the induction of ceramides, such as glucocerebrosidase (GBA) and sphingomyelin phosphodiesterase 1 (Smpd1), with 13% and 15%, respectively. The expression of AQP1, FLG and involucrin, involved in skin hydration were increased with 32%, 21%, and 75%. Another important enzyme, such as hyaluronan synthase 2 and 3 (HAS 2 and 3) found in the dermis and epidermis also contribute in keeping the skin in proper hydrated state. The activity of both, HAS 2 and 3 was increased by 23% and 126% in fibroblasts and keratinocytes. An improvement of the collagen syntheses and protection was observed by the increased production of collagen types I and III and fibronectin by 33%, 67%, and 19% [22]. A liposomal extract of whole cell suspension culture from *Malus domestica* was prepared from the Switzerland company Mibelle AG Biochemistry by incorporation of the extract in 10% lipid fraction. The obtained liposomes resulted in aqueous phase containing all the water-soluble ingredients and liposomes incorporating the oil soluble ingredients. At only 0.1% of the extract was able to stimulate the human stem cells proliferation [23]. At concentration of 0.01% was able to induce with 80% its protective activities in mammalian stem cells under exposure to UV light [56]. The extract was also able to retard the senescence in fibroblast cells (stimulated with H_2_O_2_) through the up-regulation of cyclin B1 (responsible for induction of proliferation), cyclin E1 (controls cell cycle), p53 (tumor suppressor factor), insulin-like growth factor (enhancer of cell proliferation) and heme oxygenase 1 (antioxidant enzyme) after incubation with 2% of the extract for 144 h [23].

Inflammatory processes lead to skin aging, therefore many studies have investigated the protective effects against inflammation by utilizing different in vitro or ex vivo models. The ability of *C. procera* aqueous extract to protect against skin inflammation and irritation, induced by lipopolysaccharides (LPS) and sodium dodecyl sulfate, was investigated in ex vivo human organ culture model. The extract appeared to decrease the concentrations of inflammatory cytokines interleukin (IL)-1β, IL-1α, tumor necrosis factor alpha (TNFα), and prostaglandin 2 (PGE_2_) with 3–4 folds compared to the control at 0.8 g/L concentration. The extract also improved the skin cells ability to adapt to hypoxia conditions and improve their wound healing capacity by elevating the expression of hypoxia-inducible factor 1 (HIF1) with 15%. Indication for improved cellular metabolism was observed by the increased activity of the enzyme phosphfructokinase-1 (PFK1), involved in the hydrolysis of ATP. An increase in fibronectin production by 20% indicated the effect of the extract on regeneration and healing of the skin [82].

An interesting strategy to increase extract(s) permeability through the skin offers the encapsulation in nanoliposomes. An encapsulated extract of *C. junos* displayed increased permeability of *p*-hydroxycinnamoylmalic acid by 5.8-fold when tested on an artificial PAMPA membrane [79]. The incorporation of *P. pyrifolia* aqueous extract in nanoliposomes increased its flux across the human epidermis by ca. 16 times and facilitated the in vitro wound recovery rates of keratinocytes and fibroblasts [84]. Another successful approach to increase the bioavailability and permeability of substances with low lipophilicities is to obtain their derivatives with increased lipophilic properties or their inclusion into liposomes or lipogels. The wound healing properties of the water-soluble verbascoside derived from the cell suspension of *Buddeleia davidii* were improved by the obtaining of its lipophilic semi-synthetic derivative ES2. The ES2 is considered as a novel and potent candidate for topical treatment of wounds, since this compound improved the wound closure more than 2-folds in HaCaT cells and SKH1 mice, compared to that of verbascoside at 10 μM concentration [11]. Another derivative of verbascoside, denoted as verbascoside pentapropionate, also exhibited lower hydrophilic profile and improved antioxidant activities than verbascoside [85]. In addition, the phenylpropanoids verbascoside and teupolioside, found in the extracts of *Syringa vulgaris* and *Ajuga reptans*, showed remarkable anti-inflammatory and wound healing properties, which were further correlated with the inhibition of ROS released from the whole blood leukocytes. The 56% teupoloside extract was able to prevent the skin against excessive influx of pro-inflammatory neutrophils and against oxidative damage by restoring GST activity. A 97% extract, containing verbascoside and teupoloside, was the most effective in the inhibition of monocyte chemoattractant protein-1 (MCP-1), IL-8, interferon gamma-induced protein 10 (IP-10) in the range of 1–50 μM concentration in primary cultures of human keratinocytes [27]. Along with verbascoside, another phenylethanoid glycoside, such as forsythoside B inhibited the inflammatory cytokines IL-8, MCP-1 and IP-10, stimulated by interferon gamma (INF-γ), in normal human keratinocytes [86].

The final stages in plant extract testing for potential cosmeceutical application are the clinical trials. The cream, obtained on the basis of the liposomal extract of *M. domestica* (PhytoCellTec^TM^ Malus Domestica), was tested over four weeks on 20 volunteers. The cream contained a 2% extract and resulted in significant reduction of wrinkle depth after two and four weeks, at 8% and 15% respectively [23]. The French company Naolys performed clinical trial of its product Refine ginger, derived from dedifferentiated plant cell cultures, on 22 women. The product was found to improve skin structure by 50% through pore reduction, obtained a mattifying effect by shininess reduction of 15% and sebum reduction of 19% after 28 days [18]. The *Oryza sativa* callus extract, containing mainly γ-oryzanol, vitamin E homologues and phenolic acids, also showed beneficial properties when tested on 28 volunteers, at 5% concentration, applied twice per day on facial skin for 12 weeks. The observed anti-aging effect comprised in improvement of skin moisture (75%), elasticity (88%) and whitening effect by 38% [6]. The oil-soluble extract of *R. ideaus* suspension, when applied on the skin of 20 female volunteers (0.1%, twice per day), improved the skin hydration by 16% after 14 days and 19% after 28 days [22].

## 6. Conclusions and Future Perspectives

Nowadays, plant in vitro systems are superior source of SMs or complex extracts that possess multiple beneficial activities for skin care. Due to the fast development of the cosmetic industry and recent trends in the cosmetic market, there has been elevated demand for novel and safe natural products (formulations). Biotechnological processes, based on cultivation of plant cells in vitro, are irreplaceable source of bioactive ingredients, produced under controlled conditions in the absence of contaminants and with consistent quality. A comprehensive understanding of the metabolic networks and the factors that control the biosynthesis of SMs is necessary to optimize the (bio)molecules mass production. These include fine tuning of the cultivation parameters (i.e., mixing and agitation) or nutrient medium optimization, precursor feeding or elicitation, to name a few.

Due to the changing regulations in the safety assessment of cosmetics, new in vitro methods to assess the authenticity and safety of cosmetic products have been developed, which aid our understanding of the molecular mechanisms underlying the cosmeceutical ingredients.

## Figures and Tables

**Table 1:** Small-scale production of secondary metabolites used as cosmeceuticals.

Plant Species	Culture Type	Bioreactor Volume and Type	Operational Conditions	Metabolite Production, mg/L	Ref.
Anthocyanins					
*Vitis vinifera* (L.) cv Gamay Fréaux var. Teinturier	Suspension	Stirred tank (2-L)	25 °C; agitation: 75 rpm; flow rate: 0.075–0.15 vvm; 5000 lux continuous fluorescent light	387	[33]
*V. vinifera* (L.) cv Gamay Fréaux var. Teinturier	Suspension	Stirred tank (20-L)	25 °C; agitation: 100 rpm; flow rate: 0.2 vvm; 5000 lux continuous fluorescent light	1200	[34]
*V. vinifera* cv. Bailey alicant A.	Suspension	Airlift (0.5-L)	25 °C in dark; flow rate: 80 mL/min	33	[35]
*Perilla frutescens*	Suspension	Stirred tank (2-L)	25 °C; agitation: 150 rpm; flow rate: 0.1 vvm; 27 W/m^2^ light irradiation	1650	[36]
Resveratrol					
*V. vinifera* cv. *Chasselas×Vitis berlandieri*	Suspension	Stirred tank (2-L)	23 °C in dark; agitation: 50 rpm; flow rate: 0.025 vvm	209	[30]
*V. labrusca* L	Suspension	Stirred tank (14-L)	23 °C in dark; agitation: 50 rpm; flow rate: 0.025 vvm	72	[37]
*Vitis labrusca* L	Suspension	Stirred tank (5-L)	23 °C in dark; agitation: 100 rpm; flow rate: 20.0–780.0 L/min	66	[32]
Rosmarinic acid					
*Salvia officinalis* L	Hairy roots Shoots	Nutrient sprinkle (5-L)	26 °C in dark; 40 s pump operation/50 s breaks 26 °C; 16 h/8 h light/dark; 45 s pump operation/40 s breaks	477.13 59.04	[38]
*Dracocephalum forrestii* W. W. Smith	Shoots	Nutrient sprinkle (10-L)	26 °C; 16 h/8 h light/dark; 25 s pump operation/2.5 s breaks	38.26	[16]
Chlorogenic acid					
*Dracocephalum forrestii* W. W. Smith	Shoots	Nutrient sprinkle (10-L)	26 °C; 16 h/8 h light/dark; 25 s pump operation/2.5 s breaks	0.07	[16]
*Leunorus sibiricus* L.	Hairy roots	Nutrient sprinkle (5-L)	26 °C; 40 s pump operation/1.5 min breaks	448	[39]
*Eleutherococcus koreanum* Nakai	Adventitious roots	Air lift (3-L)	22 °C in dark; flow rate: 0.1 vvm	78.22	[40]
*E. koreanum* Nakai	Adventitious roots	Bulb type (3-L)	22 °C in dark; 0.1 vvm flow rate	24.68	[41]
Caffeic acid					
*Dracocephalum forrestii* W. W. Smith	Shoots	Nutrient sprinkle (10-L)	26 °C; 16 h/8 h light/dark; 25 s pump operation/2.5 s breaks	0.07	[16]
*Leunorus sibiricus* L.	Hairy roots	Nutrient sprinkle (5-L)	26 °C; 40 s pump operation/1.5 min breaks	302	[39]
Verbascoside					
*Harpagophytum procumbens*	Suspension	Stirred tank (3-L)	26 °C in dark; agitation: 100 rpm; flow rate: 1/L min	445.44	[42]
*Harpagophytum procumbens*	Suspension	Column bioreactor with pulsed aeration (1-L)	26 °C in dark; 1/L min flow rate every 2 s	496.30	[42]
*Scutellaria alpina*	Shoots	Nutrient sprinkle (5-L)	26 °C; 40 s pump operation/2 min breaks	11.4	[43]

**Table 2:** Activities of plant cell culture extracts and their modulation activity on gene expression in skin cells in vitro models.

Plant Species and Extract Type	Concentration of the Extract, μg/mL	Gene/Protein Expression	Main Activity	Ref.
*Calotropis procera*/aqueous extract	8000	IL-1β, IL-1α, TNFα PGE_2_ inhibition HIF1 induction	Anti-inflammatory activity Hypoxia adaptation and wound healing activity	[82]
*Citrus junos*/aqueous	500	Tyrosinase inhibition Procollagen type I induction	Melanin inhibition Skin regeneration	[79]
*Hibsicus syriacus*/aqueous cell extract	20	COL I and pro-collagen I induction AQP3 and FLG induction	Collagen synthesis and protection Skin hydration	[80]
*Isodon rugosus* (Wall. ex Benth.)/80% aqueous methanol	50	MMP1, hyaluronidase, elastase inhibition SIRT-1 activation	Collagen synthesis and protection DNA repair and protection	[81]
*Lycopersicon esculentum*/aqueous extract	100	COL I and COL III induction, MMP1, MMP3 and MPP9 inhibition GADD45α and SIRT-1 induction	Collagen synthesis and protection DNA repair and protection	[21]
*Malus domestica*/liposomal extract of whole cells	100	Cyclin B1, cyclin E1 induction	Retard the signs of senescence	[23]
*Nicotiana tabacum* BY2 (*N. sylvestris*)/aqueous cell wall extract	3.6	COL I and COL III induction, pro-collagen I and III induction, MMP1, MMP3 and MPP9 inhibition GADD45α, SIRT-1 and SIRT-6 induction	Collagen synthesis and protection DNA repair and protection	[4]
*Pyrus pyrifolia* var. *culta*/aqueous extract	1000	Tyrosinase inhibition Pro-collagen I induction	Melanin inhibition Collagen synthesis	[83]
*Rubus ideaus*/oil-soluble extract	1000	GBA, Smpd1 induction AQP3, FLG, AQP3 induction COL I and III induction	Skin lipid production Skin hydration Collagen synthesis	[22]

## References

[1] Schmitz C., Fritsch L., Fischer R., Schillberg S., Rasche S. (2016). Statistical experimental designs for the production of secondary metabolites in plant cell suspension cultures. Phytochem. Lett..

[2] Maia J., Dantas T., da Costa Neto B., Borges K., Lima E., da Mata A., de Medeiros M., Pereira C. (2019). Extract of spray-dried Malay apple (*Syzygium malaccense* L.) skin. J. Food Proc. Eng..

[3] Espinosa-Leal C., Garcia-Lara S. (2019). Current methods for the discovery of new active ingredients from natural products for cosmeceutical applications. Planta Med..

[4] Apone F., Tito A., Carola A., Arciello S., Tortora A., Filippini L., Monoli I., Cucchiara M., Gibertoni S., Chrispeel M. (2010). A mixture of peptides and sugars derived from plant cell walls increases plant defense responses to stress and attenuates ageing-associated molecular changes in cultured skin cells. J. Biotechnol..

[5] Antonopoulou I., Varriale S., Topakas E., Rova U., Christakopoulous P., Faraco V. (2016). Enzymatic synthesis of bioactive compounds with high potential for cosmeceutical application. Appl. Microbiol. Biotechnol..

[6] Vichit W., Saewan N., Prinyarux T. (2018). Anti-aging efficacy of Thai red rice callus cosmetic product. J. Appl. Sci..

[7] Korkina L., Mayer W., de Luca C. (2017). Meristem plant cells as a sustainable source of redox actives for skin rejuvenation. Biomolecules.

[8] Appelhagen I., Wulff-Vester A., Wendell M., Hvoslef-Eide A.-K., Russell J., Oertel A., Martens S., Mock H.-P., Martin C., Martos A. (2018). Colour bio-factories: Towards scale-up production of anthocyanins in plant cell cultures. Metab. Eng..

[9] Rodrigues T., Amore T., Teixeira E., de Medeiros Burkert J. (2019). Carotenoid production by *Rhodotorula mucilaginosa* in batch and fed-batch fermentation using agroindustrial byproducts. Food Technol. Biotechnol..

[10] Inyai C., Boonsnongcheep P., Komaikul J., Sritularak B., Tanaka H., Putalun W. (2019). Alginate immobilization of *Morus alba* L. cell suspension cultures improved the accumulation and secretion of stilbenoids. Bioproc. Biosyst. Eng..

[11] Crivellari I., Vertuani S., Lim Y., Cervellati F., Baldisserotto A., Manfredini S., Valacchi G. (2018). ES2 as a novel verbascoside-derived compound in the treatment of cutaneous wound healing. Cosmetics.

[12] Kikowska M., Chmielewska M., Włodarczyk A., Studzińska-Sroka E., Żuchowski J., Stochmal A., Kotwicka M., Thiem B. (2018). Effect of pentacyclic triterpenoids-rich callus extract of *Chaenomeles japonica* (Thunb.) Lindl. ex Spach on viability, morphology, and proliferation of normal human skin fibroblasts. Molecules.

[13] Qu J., Zhang W., Yu X. (2011). A combination of elicitation and precursor feeding leads to increased anthocyanin synthesis in cell suspension cultures of *Vitis vinifera*. Plant. Cell Tissue Organ. Cult..

[14] Saw N., Moser C., Martens S., Franceschi P. (2017). Applying generalized additive models to unravel dynamic changes in anthocyanin biosynthesis in methyl jasmonate elicited grapevine (*Vitis vinifera* cv. Gamay) cell cultures. Hortic. Res..

[15] Jung S., Venkatesh J., Kang M.-Y., Kwon J.-K., Kang B.-C. (2019). A non-LTR retrotransposon activates anthocyanin biosynthesis by regulating a MYB transcription factor in *Capsicum annuum*. Plant. Sci..

[16] Weremczuk-Jeżyna I., Kochan E., Szymczyk P., Lisiecki P., Kuźma Ł., Grzegorczyk-Karolak I. (2019). The antioxidant and antimicrobial properties of phenol-rich extracts of *Dracocephalum forrestii* W. W. Smith shoot cultures grown in the nutrient sprinkle bioreactor. Phytochem. Lett..

[17] Lee E.-K., Jin Y.-W., Park J., Yoo Y., Hong S., Amir R., Yan Z., Elfick A., Tomlinson S., Halbritter F. (2010). Cultured cambial meristematc cells as a source of plant natural products. Nat. Biotechnol..

[18] Trehan S., Michniak-Kohn B., Beri K. (2017). Plant stem cells in cosmetics: Current trends and future directions. Future Sci..

[19] Barbulova A., Apone F., Colucci G. (2014). Plant cell cultures as source of cosmetic active ingredients. Cosmetics.

[20] Miastkowska M., Sikora E. (2018). Anti-aging properties of plant stem cell extracts. Cosmetics.

[21] Tito A., Carola A., Bimonte M., Barbulova A., Arciello S., de Laurentiis F., Monoli I., Hill J., Gilbertoni S., Colucci G. (2011). A tomato stem cell extract, containing antioxidant compounds and metal chelating factors, protects skin cells from heavy metalinduced damages. Int. J. Cosmet. Sci..

[22] Tito A., Bimonte M., Carola A., De Lucia A., Barbulova A., Tortora A., Coluccu G., Apone F. (2015). An oil-soluble extract of Rubus idaeus cells enhances hydration and water homeostasis in skin cells. Int. J. Cosmet. Sci..

[23] Schmid D., Schürch C., Blum P., Belser E., Zülli F. (2008). Plant stem cell extract for longevity of skin and hair. Int. J. Appl. Sci..

[24] Miras-Moreno B., Pedreño M., Romero L. (2019). Bioactivity and bioavailability of phytoene and strategies to improve its production. Phytochem. Rev..

[25] Jeandet P., Clément C., Tisserant L.-P., Crouzet J., Courot É. (2016). Use of grapevine cell cultures for the production of phytostilbenes of cosmetic interest. Comptes Rendus Chime..

[26] Komaikul J., Kitisripanya T., Tanaka H., Sritularak B., Putalan W. (2015). Enhanced mulberroside A production from cell suspension and root cultures of *Morus alba* using elicitation. Nat. Prod. Commun..

[27] Korkina L., Mikhal’chik E., Suprun M., Pastore S., Daltoso R. (2007). Molecular mechanisms underlying wound healing and anti-inflammatory properties of naturally occurring biotechnologically produced phenylpropanoid glycosides. Cell. Mol. Biol..

[28] Tofighi Z., Amini M., Shirzadi M., Mirhabibi H., Saeedi N., Yassa N. (2016). *Vigna radiata* as a new source for biotransformation of hydroquinone arbutin. Pharm. Sci..

[29] Khosvari E., Mousavi A., Farhadpour M., Ghashghaie J., Ghanati F., Haghbeen K. (2019). Pyrrolizidine alkaloids-free extract from the cell culture of *Lithospermum officinale* with high antioxidant capacity. Appl. Biochem. Biotechnol..

[30] Donnez D., Kim K.-H., Antoine S., Conreux A., De Luca V., Jeandet P., Clément C. (2011). Bioproduction of resveratrol and viniferins by an elicited grapevine cell culture in a 2 L stirred bioreactor. Process. Biochem..

[31] Georgiev M., Weber J. (2014). Bioreactors for plant cells: Hardware configuration and internal environment optimization as tools wider commercialization. Biotechnol. Lett..

[32] Chastang T., Pozzobon V., Taidi B., Courot E., Clément C., Pareau D. (2018). Resveratrol production by grapevine cells in fed-batch bioreactor: Experiments and modelling. Biochem. Eng. J..

[33] Aumont V., Larronde F., Richard T., Budzinski H., Decendit A., Deffieux G., Krisa S., Mérillon J.-M. (2004). Production of highly ^13^C-labeled polyphenols in *Vitis vinifera* cell bioreactor cultures. J. Biotechnol..

[34] Decendit A., Ramawat K., Waffo P., Deffieux G., Badoc A., Mérillon J.-M. (1996). Anthocyanins, catechins, condensed tannins and piceid, production in *Vitis vinifera* cell bioreactor culture. Biotechnol. Lett..

[35] Honda H., Hiraoka K., Nagamori E., Omote M., Kato Y., Hiraoka S., Kobayashi T. (2002). Enhanced anthocyanin production from grape callus in an air-lift type bioreactor using a viscous additive-supplement medium. J. Biosci. Bioengin..

[36] Zhong J.-J., Yoshida M., Fujiyama K., Seki T., Yoshida T. (1993). Enhancement of anthocyanin production by *Perilla frutescens* cells in a stirred tank bioreactor with internal light irradiation. J. Ferment. Bioeng..

[37] Nivelle L., Hubert J., Courot E., Borie N., Renault J.-H., Nuzillard J.-M., Harakat D., Clément C., Martiny L., Delmas D. (2017). Cytotoxicity of labruscol, a new resveratrol dimer produced by grapevine cell suspensions, on human skin melanoma cancer cell line HT-144. Molecules.

[38] Grzegorczyk I., Wysokińska H. (2010). Antioxidant compounds in *Salvia officinalis* L. shoot and hairy root cultures in the nutrient sprinkle bioreactor. Acta. Soc. Bot. Pol..

[39] Sitarek P., Kowalczyk T., Picot L., Michalska-Hejduk D., Bijak M., Białas A., Wielanek M., Śliwiński T., Skała E. (2018). Growth of *Leonurus sibiricus* L. roots with over-expression of AtPAP1 transcriptional factor in closed bioreactor, production of bioactive phenolic compounds and evaluation of their biological activity. Ind. Crop. Prod..

[40] Lee E.-J., Park S.-Y., Paek K.-Y. (2015). Enhancement strategies of bioactive compound production in adventitious root cultures of *Eleutherococcus koreanum* Nakai subjected to methyl jasmonate and salicylic acid elicitation through airlift bioreactors. Plant. Cell Tissue Organ. Cult..

[41] Lee E.-J., Paek K.-Y. (2012). Effect of nitrogen source on biomass and bioactive compound production in submerged cultures of *Eleutherococcus koreanum* Nakai adventitious roots. Biotechnol. Prog..

[42] Georgiev M., Ludwig-Müller J., Weber J., Stancheva N., Bley T. (2011). Bioactive metabolite production and stress-related hormones in Devil’s claw cell suspension cultures grown in bioreactors. App. Microbiol. Biotechnol..

[43] Grzegorczyk-Karolak I., Rytczak P., Bielecki S., Wysokińska H. (2017). The influence of liquid systems for shoot multiplication, secondary metabolite production and plant regeneration of *Scutellaria alpina*. Plant. Cell Tissue Organ. Cult..

[44] Rani A., Meghana R., Kush A. (2018). Squalene production in the cell suspension cultures of Indian sandalwood (*Santalum album* L.) in shake flasks and air lift bioreactor. Plant. Cell Tissue Organ. Cult..

[45] Sun D., Li C., Qin H., Zhang Q., Yang Y., Ai J. (2016). Somatic embryos cultures of *Vitis amurensis* Rupr. in air-lift bioreactors for the production of biomass and resveratrol. J. Plant. Biol..

[46] Lee E.-J., Paek K.-Y. (2012). Enhanced productivity of biomass and bioactive compounds through bioreactor cultures of *Eleutherococcus koreanum* Nakai adventitious roots affected by medium salt strength. Ind. Crop. Prod..

[47] Kovatcheva-Apostolova E., Georgiev M., Ilieva M., Skibsted L., Rødtjer A., Andersen M. (2008). Extracts of plant cell cultures of *Lavandula vera* and *Rosa damascene* as sources of phenolic antioxidants for use in foods. Eur. Food Res. Technol..

[48] Cui H.-Y., Baque A., Lee E.-J., Paek K.-Y. (2013). Scale-up of adventitious root cultures of *Echinacea angustifolia* in a pilot-scale bioreactor for the production of biomass and caffeic acid derivatives. Plant. Biotechnol. Rep..

[49] Wu C., Tang J., Jin Z., Wang M., Liu Q., Huang T., Lian M. (2018). Optimizing co-culture conditions of adventitious roots of *Echinacea pallida* and *Echinacea purpurea* in air-lift bioreactor systems. Biochem. Eng. J..

[50] Ho T.-T., Lee J.-D., Jeong C.-S., Paek K.-Y., Park S.-Y. (2017). Improvement of biosynthesis and accumulation of bioactive compounds by elicitation in adventitious root cultures of *Polygonum multiflorum*. Appl. Microbiol. Biotechnol..

[51] Cui X.-H., Niranjana H., Murthy N., Paek K.-Y. (2014). Pilot-scale culture of *Hypericum perforatum* L. adventitious roots in airlift bioreactors for the production of bioactive compounds. Appl. Biochem. Biotechnol..

[52] Wu C.-H., Murthy H., Hahn E.-J., Paek K.-Y. (2007). Large-scale cultivation of adventitious roots of *Echinacea purpurea* in airlift bioreactors for the production of chichoric acid, chlorogenic acid and caftaric acid. Biotechnol. Lett..

[53] Kobayashi Y., Akita M., Sakamoto K., Liu H., Shigeoka T., Koyano T., Kawamura M., Furuya T. (1993). Large-scale production of anthocyanin by *Aralia cordata* cell suspension culture. Appl. Microbiol. Biotechnol..

[54] Ochoa-Villarreal M., Howat S., Hong S., Jang M., Jin Y.-W., Lee E.-K., Loake G. (2016). Plant cell culture strategies for the production of natural products. Bmb Rep..

[55] Nohynek L., Bailey M., Tähtiharju J., Seppänen-Laakso T., Rischer H., Oksman-Caldenty K.-M., Puupponen-Pimiä R. (2014). Cloudberry (*Rubus chamaemorus*) cell culture with bioactive substances: Establishment and mass propagation for industrial use. Eng. Life Sci..

[56] Schürch C., Blum P., Zülli F. (2008). Potential of plant cells in culture for cosmetic application. Phytochem. Rev..

[57] Lai C., Pan H., Huang X., Fan L., Duan C., Li S. (2018). Validation of reference genes for gene expression analysis of response to anthocyanin induction in cell cultures of Vitis davidii (Rom. Caill.) Foëx. In Vitro Cell. Dev. Biol. Plant..

[58] Liu Y., Tikunov Y., Schouten R., Marcelis L., Visser R., Bovy A. (2018). Anthocyanin biosynthesis and degradation mechanisms in *Solanaceous* vegetables: A review. Front. Chem..

[59] He F., Mu L., Yan G.-L., Liang N.-N., Pan Q.-H., Wang J., Reeves M., Duan C.-Q. (2010). Biosynthesis of anthocyanins and their regulation in colored grapes. Molecules.

[60] Gao J., Li W.-B., Liu H.-F., Chen F.-B. (2019). De novo transcriptome sequencing of radish (*Raphanus sativus* L.) fleshy roots: Analysis of major genes involved in the anthocyanin synthesis pathway. Bmc Mol. Cell Biol..

[61] Liu Z., Shi M.-Z., Xie S.-Y. (2014). Regulation of anthocyanin biosynthesis in *Arabidopsis thaliana* red *pap1**-D* cells metabolically programmed by auxins. Planta.

[62] Li Y., Li H., Wang F., Li J., Zhang Y., Wang L., Gao J. (2016). Comparative transcriptome analysis reveals effects of exogenous hematin on anthocyanin biosynthesis during strawberry fruit ripening. Int. J. Genom..

[63] Jaakola L. (2013). New insights into the regulation of anthocyanin biosynthesis in fruits. Trends Plant. Sci..

[64] Piero A. (2015). The state of the art in biosynthesis of anthocyanins and its regulation in pigmented sweet oranges [(*Citrus sinensis*) L. Osbeck]. J. Agric. Food Chem..

[65] Soubeyrand E., Colombié S., Beauvoit B., Dai Z., Cluzet S., Hilbert G., Renaud C., Maneta-Peyret L., Dieuaide-Noubhani M., Mérillon J.-M. (2018). Constraint-based modeling highlights cell energy, redox status and α-ketoglutarate availability as metabolic drivers for anthocyanin accumulation in grape cells under nitrogen limitation. Front. Plant. Sci..

[66] Klimek-Chodacka M., Oleszkiewicz T., Lowder L., Qi Y., Baranski R. (2018). Efficient CRISPR/Cas9-based genome editing in carrot cells. Plant. Cell Rep..

[67] Mulinacci N., Giaccherini C., Santamaria A., Caniato R., Ferrari F., Valletta A., Vincieri F., Pasqua G. (2008). Anthocyanins and xanthones in the calli and regenerated shoots of *Hypericum perforatum* var. *angustifolium* (sin. Fröhlich) Borkh. Plant. Physiol. Biochem..

[68] Nadeem M., Abbasi B., Younas M., Ahmad W., Zahir A., Hano C. (2019). LED-enhanced biosynthesis of biologically active ingredients in callus cultures of *Ocimum basilicum*. Photochem. Photobiol..

[69] Biswas T., Singh M., Mathur A., Mathur A. (2015). A dual purpose cell line of an Indian congener of ginseng-*Panax sikkimensis* with distinct ginsenoside and anthocyanin production profiles. Protoplasma.

[70] Tavares S., Vesentini D., Fernandes J., Ferreira R., Laureano O., Ricardo-Da-Silva J., Amâncio S. (2013). *Vitis vinifera* secondary metabolism as affected by sulfate depletion: Diagnosis through phenylpropanoid pathway genes and metabolites. Plant. Physiol. Biochem..

[71] Yin Y., Borges G., Sakuta M., Crozier A., Ashihara H. (2012). Effect of phosphate deficiency on the content and biosynthesis of anthocyanins and the expression of related genes in suspension-cultured grape (*Vitis* sp.) cells. Plant. Physiol. Biochem..

[72] Tassoni A., Durante L., Ferri M. (2012). Combined elicitation of methyl-jasmonate and red light on stilbene and anthocyanin biosynthesis. J. Plant. Physiol..

[73] Davis G., Ananda A., Krastanova S., Sutton S., Ochieng J., Leong S., Tsolova V. (2012). Elevated gene expression in chalcone synthase enzyme suggests an increased production of flavonoids in skin and synchronized red cell cultures of North American native grape berries. DNA Cell Biol..

[74] Oglesby L., Ananga A., Obuya J., Ochieng J., Cebert E., Tsolova V. (2016). Anthocyanin accumulation in muscadine berry skins is influenced by the expression of the MYB transcription factors, *MybA1*, and *MYBCS1*. Antioxidants.

[75] Wang H., Wang W., Huang W., Xu H. (2017). Effect of salicylic acid on the gene transcript and protein accumulation of flavonoid biosynthesis-related enzymes in *Vitis vinifera* cell suspension cultures. Hortic. Sci..

[76] Martins V., Garcia A., Costa C., Sottomayor M., Gerós H. (2018). Calcium- and hormone-driven regulation of secondary metabolism and cell wall enzymes in grape berry cells. J. Plant. Physiol..

[77] Sinilal B., Ovadia R., Nissim-Levi A., Perl A., Carmeli-Weissberg M., Oren-Shamir M. (2011). Increased accumulation and decreased catabolism of anthocyanins in red grape cell suspension culture following magnesium treatment. Planta.

[78] Matos M., Romero-Díez R., Álvarez A., Bronze M., Rodríguez-Rojo S., Mato R., Cocero M., Matias A. (2019). Polyphenol-rich extracts obtained from winemaking waste streams as natural ingredients with cosmeceutical potential. Antioxidants.

[79] Adhikari D., Panthi V., Pangeni R., Kim H., Park J. (2017). Preparation, characterization, and biological activities of topical anti-aging ingredients in a *Citrus junos* callus extract. Molecules.

[80] Di Martino O., Tito A., De Lucia A., Cimmino A., Cicotti F., Apone F., Colucci G., Calabrò V. (2017). *Hibiscus syriacus* extract from an established cell culture stimulates skin wound healing. Biomed. Res. Int..

[81] Abbasi B., Siddiquah A., Tungmunnithum D., Bose S., Younas M., Garros L., Drouet S., Giglioli-Guivarćh N., Hano C. (2019). *Isodon rugosus* (Wall. ex Benth.) Codd in vitro cultures: Establishment, phytochemical characterization and in vitro antioxidant and anti-aging activities. Int. J. Mol. Sci..

[82] Portugal-Cohen M., Ish-Shalom E., Mallon R., Corral P., Michoux F., Ma’or Z. (2018). Apple of Sodom (*Calatropis procera*) callus extract, a novel skincare active and its biological activity in skin models when combined with Dead Sea water. J. Cosmet. Derm. Sci. Appl..

[83] Kim H., Park J. (2017). Anti-aging activities of *Pyrus pyrifolia* var culta plant callus extract. Trop. J. Pharm. Res..

[84] Park D., Adhikari D., Pangeni R., Panthi V., Kim H., Park J. (2018). Preparation and characterization of callus extract from *Pyrus pyrifolia* and investigation of its effects on skin regeneration. Cosmetic.

[85] Vertuani S., Beghelli E., Scalambra E., Malisardi G., Copetti S., Dal Toso R., Baldisserotto A., Manfredini S. (2011). Activity and stability studies of verbascoside, a novel antioxidant, in dermo-cosmetic and pharmaceutical topical formulations. Moleclues.

[86] Georgiev M., Pastore S., Lulli D., Alipieva K., Kostyuk V., Potapovich A., Panetta M., Korkina L. (2012). *Verbascum xanthophoeniceum*-derived phenylethanoid glycosides are potent inhibitors of inflammatory chemokines in dormant and interferon-gamma-stimulated human keratinocytes. J. Ethnopharmacol..

